# Case Report: Aplastic anemia associated with parvovirus B19 infection following neoadjuvant pembrolizumab-based chemoimmunotherapy for triple-negative breast cancer

**DOI:** 10.3389/fonc.2026.1834150

**Published:** 2026-07-10

**Authors:** Jan Kindl, Dominika Svobodová, Martin Matějů, Martina Zimovjanová, Michal Vočka, Petra Kovaříková, Zuzana Bielčiková

**Affiliations:** 1 Department of Oncology, General University Hospital in Prague and First Faculty of Medicine, Charles University in Prague, Prague, Czechia; 2 Hematology, First Department of Medicine, General University Hospital and First Faculty of Medicine, Charles University Prague, Prague, Czechia

**Keywords:** aplastic anemia, immune checkpoint inhibitors, immune-related adverse events, intravenous immunoglobulins, pancytopenia, parvovirus B19, pembrolizumab, triple-negative breast cancer

## Abstract

Immune checkpoint inhibitors are increasingly incorporated into curative treatment strategies for early-stage triple-negative breast cancer. Although hematologic immune-related adverse events are rare, they may be severe and difficult to distinguish from other causes of bone marrow failure. We report a 54-year-old woman with triple-negative breast cancer treated with neoadjuvant pembrolizumab plus chemotherapy according to the KEYNOTE-522 regimen. After breast-conserving surgery and achievement of a pathologic complete response, she developed a rapidly progressive severe pancytopenia (grade 4 febrile neutropenia, grade 4 thrombocytopenia and grade 3 anemia) in the early postoperative period. Bone marrow biopsy showed generalized bone marrow suppression. An immune-related hematologic toxicity was suspected; high-dose corticosteroids were initiated. However, no hematologic response was observed. Further tests detected ongoing parvovirus B19 infection (positive plasma viral DNA and IgM and IgG serology). Intravenous immunoglobulin therapy was therefore initiated, resulting in rapid hematologic recovery. Pembrolizumab was discontinued, and the patient subsequently completed adjuvant radiotherapy. At a 20-month follow-up, she remained free of disease recurrence. This case highlights the importance of broad diagnostic evaluation of severe cytopenias after pembrolizumab-based chemoimmunotherapy and underscores the need to distinguish infectious bone marrow suppression from primary immune-related hematologic toxicity, as this distinction may directly influence management.

## Introduction

Immune checkpoint inhibitors have fundamentally transformed therapeutic options for many types of cancer. Initially established in the palliative setting, immunotherapy has progressively expanded into curative treatment strategies. Immunotherapy has also become an integral component of therapy for early-stage triple-negative breast cancer (defined as a tumor lacking expression of estrogen and progesterone receptors and without HER2 amplification). In the KEYNOTE-522 trial (1), the combination of pembrolizumab with chemotherapy in both the neoadjuvant and adjuvant settings for triple-negative breast cancer resulted in a significant increase in pathological complete response rates as well as improved long-term outcomes.

However, immune checkpoint inhibitors introduce a distinct spectrum of immune-related adverse events (irAEs), which can affect virtually any organ system. The most common toxicities affect the skin, endocrine glands, and gastrointestinal tract. Less frequently observed are hepatic dysfunction, pneumonitis, musculoskeletal toxicities and renal, neurologic, or cardiac toxicities (2). A similar distribution of adverse events is described in the KEYNOTE-522 trial as well (1, 3).

Among all the adverse events, hematologic toxicities associated with immunotherapy are considered rare, with an incidence rate generally below 5%, typically ranging from approximately 0.5% to 3.6% (2, 4–7). The spectrum of hematologic manifestations includes autoimmune hemolytic anemia, immune thrombocytopenic purpura, isolated and combined cytopenias, hemophagocytic lymphohistiocytosis, and severe bone marrow suppression such as aplastic anemia (4–8).

Although uncommon, hematologic adverse events can have a severe and potentially life-threatening clinical course. Due to their low incidence, these complications may pose a diagnostic challenge in routine clinical practice, leading to delays in initiating appropriate treatment. Their management is further complicated by the limited data available from clinical trials. Consequently, current management strategies are largely based on small case series and individual case reports (2, 4–7).

We present the case of a patient with triple-negative breast cancer treated with standard neoadjuvant chemoimmunotherapy who developed severe aplastic anemia in the early postoperative period. To our knowledge, severe aplastic anemia occurring in the early postoperative period after neoadjuvant pembrolizumab-based chemoimmunotherapy in the setting of concomitant parvovirus B19 infection has not been previously described. This case illustrates the complex interplay between infection and immune checkpoint inhibition. It also underscores the importance of carefully distinguishing infection-triggered bone marrow suppression from primary immune-related hematologic toxicity. This distinction may have direct implications for therapeutic decision-making.

## Case description

A 54-year-old female patient with no significant comorbidities had previously undergone surgery for a small right-sided hormone receptor–positive breast cancer in 2019. She subsequently initiated adjuvant endocrine therapy with tamoxifen without indication for chemotherapy. In August 2023, while still undergoing endocrine therapy, the patient discovered a lump in the contralateral breast. Imaging studies classified the finding as cT1cN1 (1 positive lymph node) M0. Core biopsy confirmed triple-negative breast cancer with a Ki-67 proliferative index of 70%.

**Figure 1:**
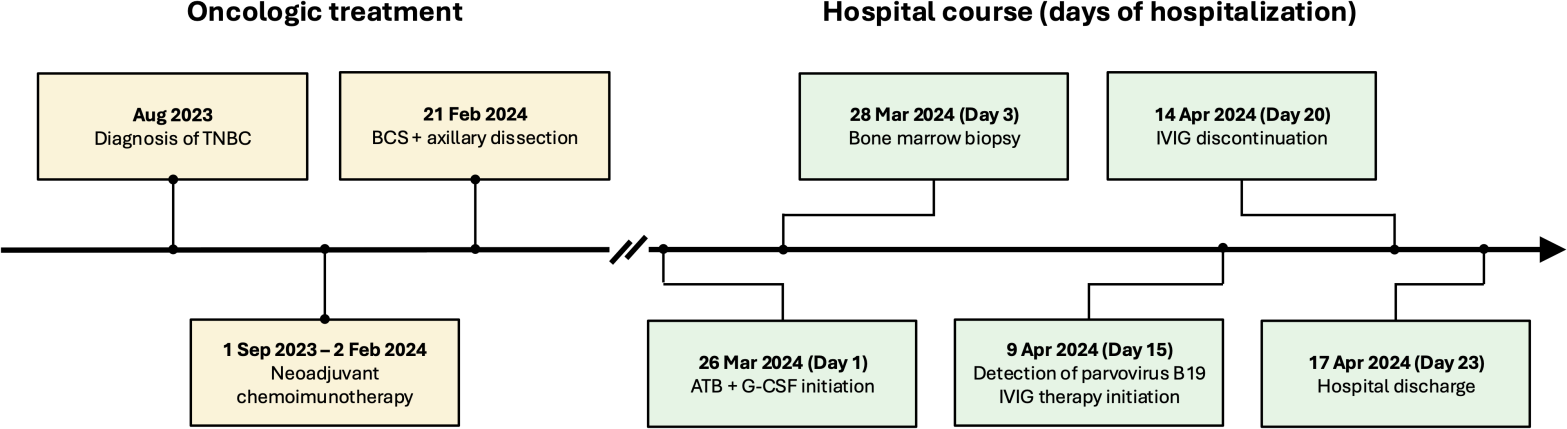
Timeline of oncologic treatment and hospital course. The image summarizes key events from the diagnosis of TNBC, through neoadjuvant pembrolizumab-based chemoimmunotherapy and breast-conserving surgery with axillary dissection, to the onset of severe hematologic toxicity. Subsequent diagnostic and therapeutic interventions are depicted, including bone marrow biopsy, detection of parvovirus B19, initiation of ATB and G-CSF, administration and discontinuation of IVIG, and hospital discharge. TNBC, triple-negative breast cancer; BCS, breast-conserving surgery; ATB, antibiotic therapy; G-CSF, granulocyte colony-stimulating factor; IVIG, intravenous immunoglobulin.

According to the KEYNOTE-522 protocol (1), the patient underwent neoadjuvant chemotherapy consisting of carboplatin and paclitaxel (carboplatin AUC 1.5 weekly and paclitaxel 80 mg/m² weekly) followed by doxorubicin and cyclophosphamide (doxorubicin 60 mg/m² and cyclophosphamide 600 mg/m² every 3 weeks), in combination with pembrolizumab (200 mg every 3 weeks). Overall, the neoadjuvant treatment was well tolerated, except for a tendency toward neutropenia. According to the Common Terminology Criteria for Adverse Events (CTCAE), version 5.0 (9), Grade 3 neutropenia occurred after cycle 1, day 15 of carboplatin plus paclitaxel. Starting with the second cycle, prophylactic granulocyte colony-stimulating factor (G-CSF), pegfilgrastim (6 mg), was administered after each cycle of chemotherapy, and neutrophil counts did not subsequently fall below 1.5 × 10^9^/L. The last cycle of neoadjuvant therapy was administered on February 2, 2024 (**Figure 1**).

Breast-conserving surgery with left axillary dissection was subsequently performed on February 21, 2024. Pathological examination demonstrated a pathologic complete response (pCR; ypT0 ypN0). Given the pCR, the patient was scheduled to continue treatment with adjuvant pembrolizumab monotherapy, as recommended by current guidelines (2).

A routine blood test performed on March 22, 2024, one month after surgery, revealed grade 3 neutropenia (absolute neutrophil count 0.68 × 10^9^/L). Adjuvant immunotherapy was planned at this time but was not administered due to neutropenia. Three days later, a repeat blood test showed complete absence of neutrophils (0,0 x 10^9^/L). In the absence of clinical signs of infection or elevated inflammatory markers, pegfilgrastim (6 mg) was administered on an outpatient basis. However, the following day, the patient developed a fever and was admitted to the hospital with febrile neutropenia. The patient was placed in protective isolation.

**Figure 2:**
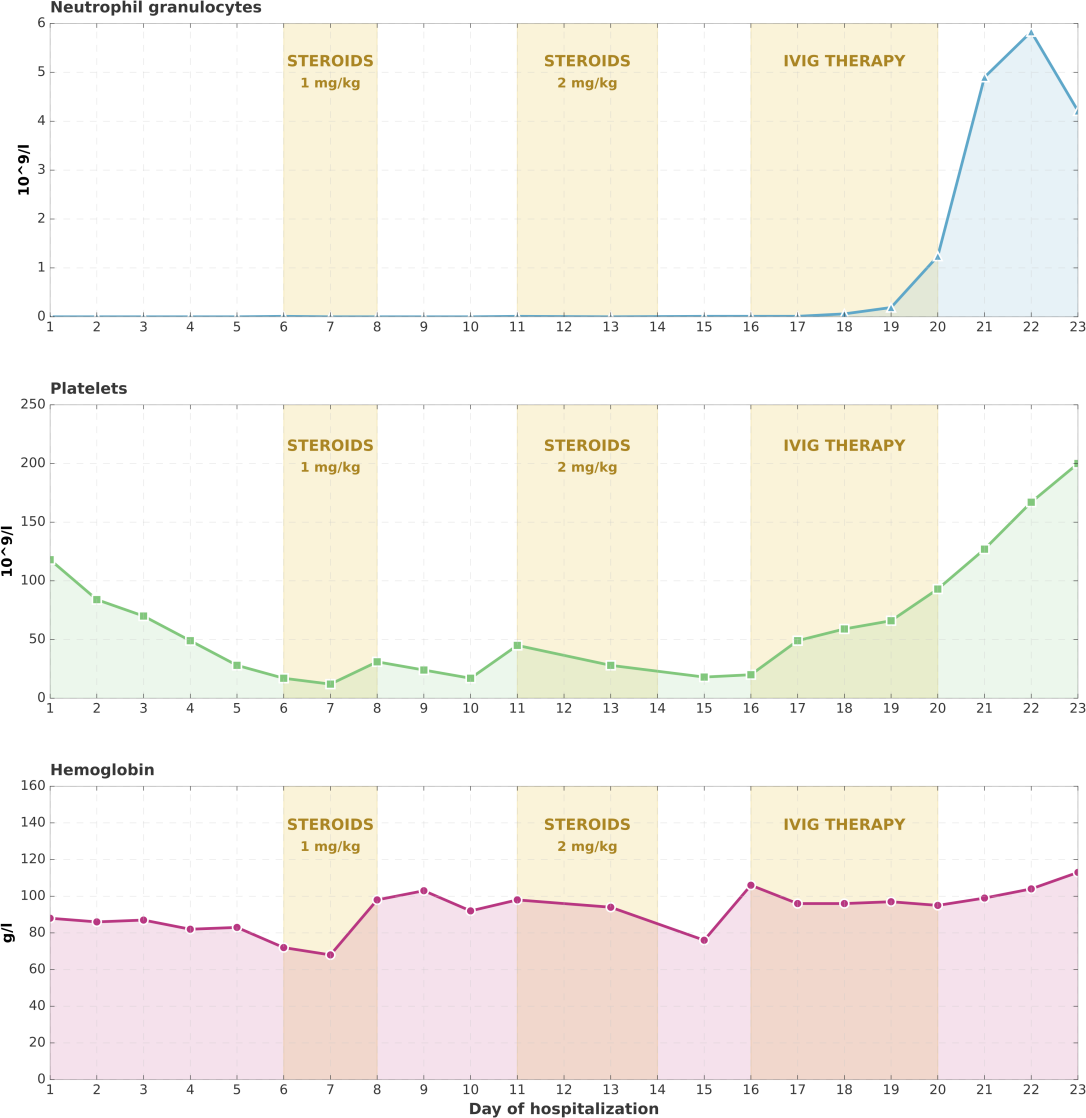
Clinical course of hematologic parameters during hospitalization. Hemoglobin level, absolute neutrophil count, and platelet count are shown over time. A profound decline in all three cell lines is observed, consistent with severe pancytopenia refractory to high-dose corticosteroid therapy, followed by gradual recovery after initiation of intravenous immunoglobulin therapy. IVIG, intravenous immunoglobulin.

Empirical antibiotic therapy with piperacillin/tazobactam and amikacin was initiated, and treatment with G-CSF was continued using daily filgrastim. Nevertheless, the neutrophil count did not increase (**Figure 2**). Thrombocytopenia subsequently developed and progressed into grade 4, accompanied by grade 3 anemia. On the third day of hospitalization, a bone marrow biopsy was performed. Biopsy demonstrated a severely hypocellular marrow with trilineage hypoplasia and approximately 10% cellularity, markedly reduced relative to the expected age-adjusted cellularity. Granulopoiesis was significantly suppressed with a left shift and predominance of immature precursor cells, while mature neutrophils were absent. Myeloblasts accounted for less than 5% of nucleated cells. Megakaryocytes were reduced in number but without significant atypia. No malignant infiltration, hemophagocytosis, or overt morphologic dysplasia was identified. Flow cytometry of both peripheral blood and bone marrow did not demonstrate leukemia or lymphoproliferative disease. Although flow cytometric findings raised limited suspicion for myelodysplastic syndrome, the overall morphologic and immunophenotypic findings were interpreted as secondary reactive marrow suppression. At the nadir, the patient developed profound pancytopenia with an absolute neutrophil count of 0.00 × 10^9^/L, platelet count of 12 × 10^9^/L, and markedly decreased absolute reticulocyte count (0.023 × 10¹²/L). Together with the severely hypocellular bone marrow, these findings fulfilled formal criteria for severe aplastic anemia according to the modified Camitta classification (10, 11).

Treatment with hematopoietic growth factors continued. Due to the consideration of an immune-related hematologic adverse event, high-dose glucocorticoids were initiated despite the ongoing infection. Methylprednisolone was administered for three days at 1 mg/kg, followed by four days at 2 mg/kg. Due to rising inflammatory markers, antibiotic therapy was escalated to meropenem in combination with linezolid. The patient received multiple transfusions of red blood cells and platelets.

On the 15th day of hospitalization, the patient was transferred to the hematology department due to persistent grade 4 pancytopenia. An extensive laboratory panel was performed (**Table 1**), including screening for viral infections, autoimmune disorders, and nutritional deficiencies. The results revealed positive parvovirus B19 DNA in the plasma, as well as IgM and IgG antibodies, consistent with an ongoing parvovirus B19 infection. Other viral tests [Human Immunodeficiency Virus (HIV), hepatitis viruses, Cytomegalovirus (CMV), and Human Herpesvirus 6 (HHV-6)] were negative, and autoimmune screening did not reveal any abnormalities. Intravenous immunoglobulin therapy (IVIG) was administered at a dose of 0.4 g/kg/day over 5 consecutive days.

**Table 1:** Diagnostic panel for aplastic anemia performed on April 9, 2024 (day 15 of the hospitalization).

Infectious work-up	
**Parvovirus B19 IgM**	**positive (index 9,25)**
**Parvovirus B19 IgG**	**positive (>150,00 IU/mL)**
**Parvovirus B19 PCR**	**positive (14,250 copies/mL)**
HIV serology	negative
HBV, HCV serology	negative
CMV PCR	negative
EBV serology	consistent with past infection
HHV-6 PCR	negative
Autoimmune work-up	
ANA/ENA/ANCA	negative
Nutritional evaluation	
Vitamin B12	mildly decreased (74 pmol/L)
Folate	mildly decreased (2,5 µg/L)
Ferritin	mildly increased (473,8 µg/L)
Inflammatory markers	
CRP	significantly increased (313,2mg/L)
Procalcitonin	normal value (0,25 µg/L)
Fibrinogen	mildly increased (6,27 g/L)

Parvovirus B19 IgM, IgG and PCR results indicating parvovirus B19 infection in bold.

A prompt hematologic response was observed following the initiation of IVIG therapy. The following day, the first increase in platelet count was noted, followed by gradual neutrophil recovery two days later. On day 20 of hospitalization, neutrophil and platelet counts improved to a grade 1 level and IVIG therapy was discontinued. Over the next few days, complete recovery of all hematologic parameters was achieved, except for persistent grade 1 anemia. After 23 days of hospitalization, the patient was discharged with normal blood counts and there was no need for further supportive therapy. Follow-up quantitative PCR testing of parvovirus B19 after IVIG therapy was not performed.

Due to the suspected association with immune checkpoint inhibitor therapy, pembrolizumab was discontinued based on a decision of a multidisciplinary team involving oncologists and hematologists. The patient subsequently underwent adjuvant radiotherapy and remains under regular oncologic follow-up. At the most recent follow-up visit in December 2025, approximately 20 months after the episode of pancytopenia, there was no evidence of disease recurrence. White blood cell and platelet counts were within the normal range, except for persistent grade 1 anemia.

## Discussion

This report describes a patient with triple-negative breast cancer who developed severe aplastic anemia shortly after completing neoadjuvant, pembrolizumab-based chemoimmunotherapy. The clinical course was characterized by progressive pancytopenia with grade 4 neutropenia that persisted for 20 days and marked bone marrow suppression. During the diagnostic evaluation, parvovirus B19 infection was identified through the detection of viral DNA in plasma, as well as positive IgM and IgG antibodies. After initiation of IVIG therapy, the patient experienced rapid hematologic recovery, supporting parvovirus B19-associated marrow suppression as a major contributor to the clinical course.

Parvovirus B19 infection is usually a childhood disease associated with erythema infectiosum. However, the virus exhibits a strong tropism for erythroid progenitor cells and can therefore suppress erythropoiesis. Diagnosis is usually made by detecting viral DNA in blood together with serological evidence of infection (12, 13). In rare cases, infection may lead to broader suppression of hematopoiesis affecting multiple cell lineages (14). Such presentations have predominantly been described in immunocompromised individuals, including transplant recipients, patients with congenital immunodeficiencies, or those receiving immunosuppressive therapy (15–17).

Parvovirus B19 infection has also been reported in patients receiving immune checkpoint inhibitors. In most published cases, however, the infection manifests as pure red cell aplasia rather than global bone marrow failure. For instance, Pallotta et al. reported on a patient treated with atezolizumab who developed parvovirus B19-associated pure red cell aplasia requiring treatment with intravenous immunoglobulins. Unlike the present case, the hematologic toxicity in that report was limited to the erythroid lineage (18).

Our patient represented a potentially vulnerable host, having received an intensive six-month course of chemotherapy combined with immune checkpoint inhibition. Although transient myelosuppression is commonly associated with cytotoxic chemotherapy, profound and prolonged pancytopenia with features of severe aplastic anemia occurring one month after treatment is unusual. The administration of pembrolizumab may have contributed to the clinical course, as PD-1 blockade profoundly modulates cellular immunity and can lead to immune-related adverse events.

Hematologic immune-related toxicities associated with PD-1/PD-L1 inhibitors are rare, and their true incidence is likely underestimated (4–6). Aplastic anemia is one of the most severe manifestations and has been reported in recent case series and pharmacovigilance analyses (19).

Given the severity of marrow failure and the potential contribution of immune-related hematologic toxicity, permanent discontinuation of pembrolizumab was recommended after multidisciplinary discussion involving oncology and hematology specialists. Rechallenge was not pursued due to the life-threatening nature of the event, the uncertainty regarding the relative contribution of PD-1 blockade, the concomitant parvovirus B19 infection, and the achievement of pathologic complete response after neoadjuvant therapy.

In the present case, the lack of response to corticosteroid therapy together with the rapid hematologic recovery following intravenous immunoglobulin treatment strongly supports the hypothesis of parvovirus B19–associated bone marrow failure. IVIG is the standard treatment for parvovirus B19 infection in immuno-compromised patients, often resulting in rapid hematologic recovery (10). The clinical presentation was likely multifactorial, with prior cytotoxic therapy, persistent immune dysregulation, and subsequent parvovirus B19 infection acting synergistically. Immune checkpoint inhibition may have contributed to immune dysregulation and susceptibility to severe marrow suppression in the setting of active parvovirus B19 infection; however, its precise contribution remains difficult to determine.

This case therefore highlights the importance of considering infectious etiologies, including parvovirus B19 infection, when evaluating severe cytopenias that occur during or after immune checkpoint inhibitor therapy.

## Conclusion

Although the precise contribution of immune checkpoint inhibition cannot be definitively determined, the rapid hematologic recovery following IVIG therapy together with positive parvovirus B19 PCR and serology supports parvovirus-associated marrow suppression as a major contributor to the clinical course. The overall presentation was likely multifactorial in the setting of recent intensive chemotherapy, PD-1 blockade, and active viral infection. This case highlights the importance of comprehensive diagnostic evaluation for patients experiencing severe hematologic complications during modern oncologic therapy.

## Patient perspective

After achieving pathological complete response, the patient initially felt relieved and optimistic about her prognosis. However, approximately one month later she required prolonged hospitalization due to severe complications described in this case report. The lack of clinical improvement during this period significantly impacted her psychologically, so supportive care was provided, including consultations with the palliative care team.

## Data Availability

The raw data supporting the conclusions of this article will be made available by the authors, without undue reservation.
